# Contribution of Chondroitin Sulfate A to the Binding of Complement Proteins to Activated Platelets

**DOI:** 10.1371/journal.pone.0012889

**Published:** 2010-09-23

**Authors:** Osama A. Hamad, Per H. Nilsson, Maria Lasaosa, Daniel Ricklin, John D. Lambris, Bo Nilsson, Kristina Nilsson Ekdahl

**Affiliations:** 1 Division of Clinical Immunology, Rudbeck Laboratory C5, Uppsala University, Uppsala, Sweden; 2 School of Natural Sciences, Linnaeus University, Kalmar, Sweden; 3 Department of Pathology and Laboratory Medicine, University of Pennsylvania, Philadelphia, Pennsylvania, United States of America; University of California Merced, United States of America

## Abstract

**Background:**

Exposure of chondroitin sulfate A (CS-A) on the surface of activated platelets is well established. The aim of the present study was to investigate to what extent CS-A contributes to the binding of the complement recognition molecule C1q and the complement regulators C1 inhibitor (C1INH), C4b-binding protein (C4BP), and factor H to platelets.

**Principal Findings:**

Human blood serum was passed over Sepharose conjugated with CS-A, and CS-A-specific binding proteins were identified by Western blotting and mass spectrometric analysis. C1q was shown to be the main protein that specifically bound to CS-A, but C4BP and factor H were also shown to interact. Binding of C1INH was dependent of the presence of C1q and then not bound to CS-A from C1q-depleted serum. The specific interactions observed of these proteins with CS-A were subsequently confirmed by surface plasmon resonance analysis using purified proteins. Importantly, C1q, C4BP, and factor H were also shown to bind to activated platelets and this interaction was inhibited by a CS-A-specific monoclonal antibody, thereby linking the binding of C1q, C4BP, and factor H to exposure of CS-A on activated platelets. CS-A-bound C1q was also shown to amplify the binding of model immune complexes to both microtiter plate-bound CS-A and to activated platelets.

**Conclusions:**

This study supports the concept that CS-A contributes to the binding of C1q, C4BP, and factor H to platelets, thereby adding CS-A to the previously reported binding sites for these proteins on the platelet surface. CS-A-bound C1q also seems to amplify the binding of immune complexes to activated platelets, suggesting a role for this molecule in immune complex diseases.

## Introduction

Glycosaminoglycans (GAG) are important structures in the extracellular matrix (ECM). Many GAGs are attached directly to cell membrane proteins and facilitate the binding of soluble proteins to the surface. Well-known GAGs include heparin, heparan sulfate, dermatan sulfate, and chondroitin sulfate [1].

Chondroitin sulfate (CS) is a GAG that consists of an anionic linear, unbranched polysaccharide of alternating disaccharide units of glucuronic acid and N-acetylgalactosamine, connected to a protein core via a tetrasaccharide linker [2]. Although conventionally viewed as important because of its structural role in the extracellular matrix, CS has recently received growing attention because of its other cellular functions, such as in cell communication [3], [4]. The sulfation pattern, deacetylation, and epimerization of the structure create diversity among the CS family and are critical for the specific activity of its individual members [4]. In mammals, the galactosamine unit is most often monosulfated at position C-4 (as in the case of CS-A) or C-6 (as in CS-C) [5]. In addition to monosulfated CS-A and CS-C, other forms of CS have been described, such as CS-D and CS-E, which both are disulfated [5]. Dermatan sulfate, formerly known as CS-B, is often described together with CS but differs more radically from the other forms of CS, mainly because of its frequent epimerization of the glucoronic acid to iduronic acid [6]. CS is the most abundant GAG in human plasma (70–80% of all GAGs), with CS-A representing half of this fraction and the remainder being non-sulfated [5].

A number of cell types express CS on their surfaces, including neurons, glial cells and platelets [7]. The fact that CS-A represents the main GAG in platelets has been well established by both biochemical and histologic techniques [8], [9]. Rapid release of CS-A from platelets has been shown to occur in response to a variety of agonists, including ADP, collagen, adrenalin, and thrombin, resulting in a rise in plasma CS-A by up to 2 µg/mL within 3 min after activation [10]. CS-A has been implicated to be localized in the platelet α-granules [10], [11], [12], and has been shown to be exposed on the surface of platelets after activation [9]. The CS-A present in platelets, unlike that in blood plasma, is fully sulfated, and its average molecular mass has been estimated to be approximately 28 kDa [8]. An over-sulfated form of CS was recently described to be contaminating commercial heparin preparations. These heparin preparations caused fatal anaphylatoxic reactions after injection/infusion due to the over-sulfated CS which activated both the complement and the contact systems [13].

We have previously shown that CS-A released from activated platelets activates the complement system in the fluid phase [14]. C1q was identified as the recognition molecule, since it bound to CS-A in high amounts. Complement activation was abolished when C1q-depleted serum was used. We have also shown that platelets activated with the thrombin receptor activating peptide (TRAP) expose CS-A and bind complement components C1q, C4, C3, and C9 [15]. TRAP acts as a tethered ligand for the thrombin receptor PAR-1 and is able to cause full receptor activation in the absence of thrombin [16], [17]. However, the binding of complement proteins is independent of complement activation, and inhibition of complement at the stage of C1q or C3 does not affect the binding of the complement components. This suggests that the complement system is stringently regulated on the platelet surface, both regarding initiation and amplification.

In previous studies, we have found a very high avidity of C1q for CS-A, which is reflected in the relative inability of soluble CS-A to compete with the binding of C1q to surface-conjugated CS-A. We also observed that high amounts of C3 bound in a non-activated from, as C3(H_2_O) which initiated speculations regarding the impact of soluble complement inhibitors at the platelet surface. In the present study, we have investigated the binding to both immobilized and platelet-exposed CS-A of C1q and of complement-regulating proteins known to interact with GAGs. We also investigated the functional capacity of CS-A-bound C1q with regard to the binding of model immune complexes to the platelet surface.

## Results

### Binding of serum proteins to Sepharose-bound CS-A

**Figure 1:**
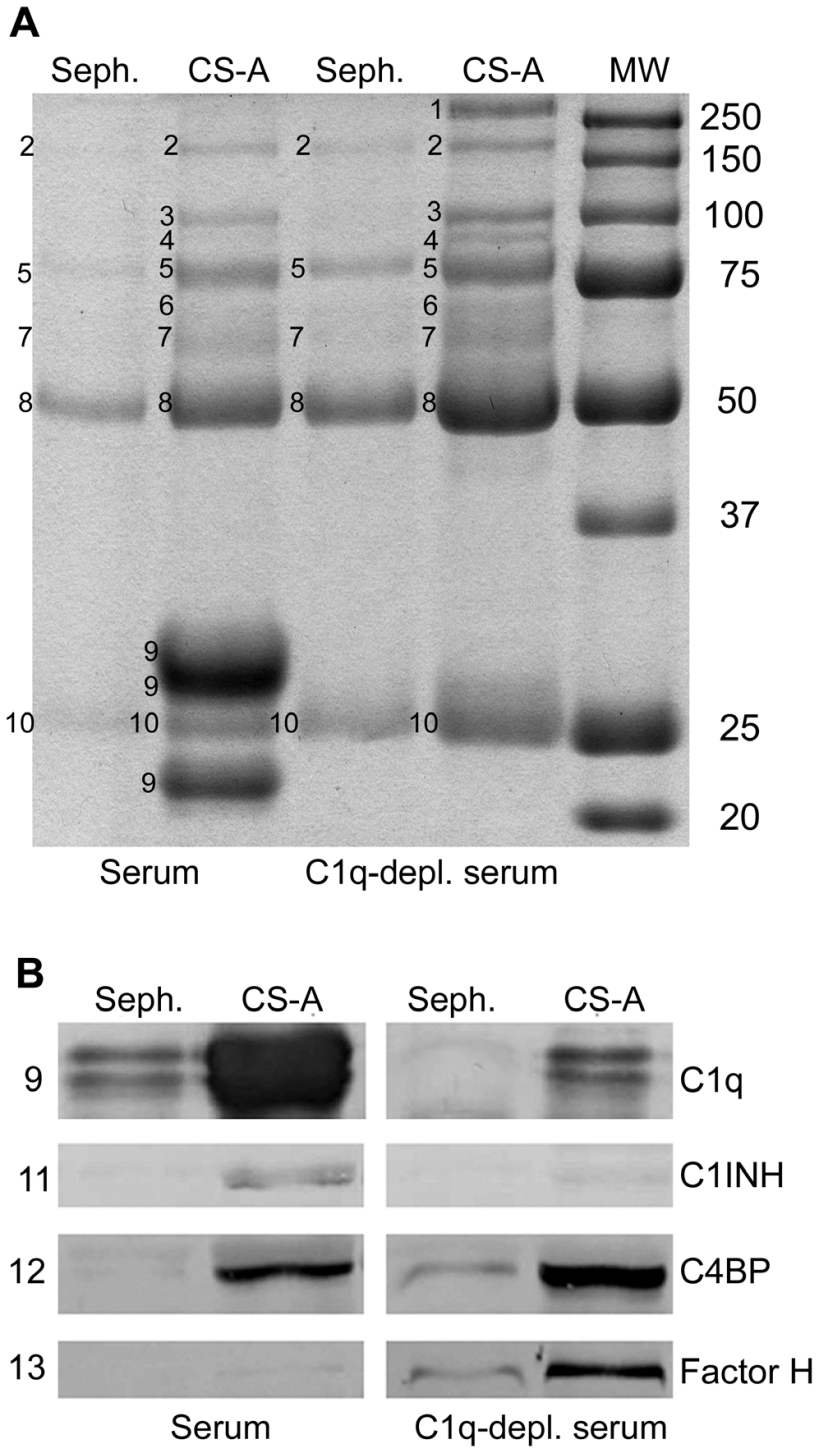
Binding of proteins to CS-A-Sepharose and control Sepharose. Proteins from serum and C1q-depleted serum that bound to the CS-A affinity (CS-A) and control (Sepharose) columns were eluted with increasing salt concentrations; the eluted proteins were concentrated, subjected to SDS-PAGE, and visualized on a gel (A) using Coomassie staining or by reaction with specific antibodies in Western blotting (B). The numbered bands in A were cut out and identified by mass spectrometry; the identification was further verified by Western blot analysis.

Serum or C1q-depleted serum was passed over Sepharose conjugated with CS-A or over a control Sepharose column without CS-A. After washing, the bound serum proteins were eluted with increasing salt concentrations. The eluted proteins were concentrated and then run on sodium dodecyl sulfate-polyacrylamide gel electrophoresis (SDS-PAGE) under reducing conditions, and the polypeptide bands were identified by mass spectrometric analysis and Western blotting (Fig. 1). Immunoglobulin-derived chains (μ, α, γ, and κ) from serum sufficient and depleted of C1q were shown to bind to both the control and CS-A conjugated columns (Fig. 1 A). The polypeptide bands with the strongest intensity that were specific for the CS-A conjugated Sepharose were associated with C1q. In control experiments it was demonstrated that the C1q binding was not dependent on bound IgG since equivalent binding of C1q occurred from IgG depleted serum as compared to intact serum (data not shown). Additional bands specifically binding to CS-A and visualized on the gels were identified as fibronectin, thrombospondin, plasminogen, thrombin-antithrombin complex and human serum albumin. When we characterized the binding of the complement regulators C1INH, C4BP, and factor H by Western blotting (Fig. 1B), we found that in the presence of C1q, C1INH, factor H and C4BP bound to the column, but after C1q depletion, binding of C1INH was no longer seen, suggesting that the protein bound to CS-A in association with C1q. Factor H and C4BP bound with higher intensity in the absence of C1q. Identified proteins are listed in Table 1.

**Table 1:** Proteins identified in CS-A and Sepharose eluate after application of serum and C1q-depleted serum.

Protein	Mass spectrometry	Western blot
1. Fibronectin	√	√
2. Thrombospondin	√	n.d.
3. Plasminogen	√	n.d.
4. Thrombin-antithrombin complex	√	√
5. Ig μ	√	√
6. Human serum albumin	√	√
7. Ig α	√	√
8. Ig γ	√	√
9. C1q, chain A,B,C	√	√
10. Ig κ	√	n.d.
11. C1INH	-	√
12. C4BP	-	√
13. Factor H	-	√

Proteins 1–10 were visualized on a gel (Fig. 1A) by Commassie blue staining and identified with mass spectrometric analysis. These proteins were also identified with western blotting, except for thrombospondin, plasminogen and Ig κ chains that were not determined (n.d.). C1INH, C4BP and factor H were identified with western blotting (Fig 1B).

### Evaluation of the binding of complement proteins to immobilized CS-A by surface plasmon resonance

**Figure 2:**
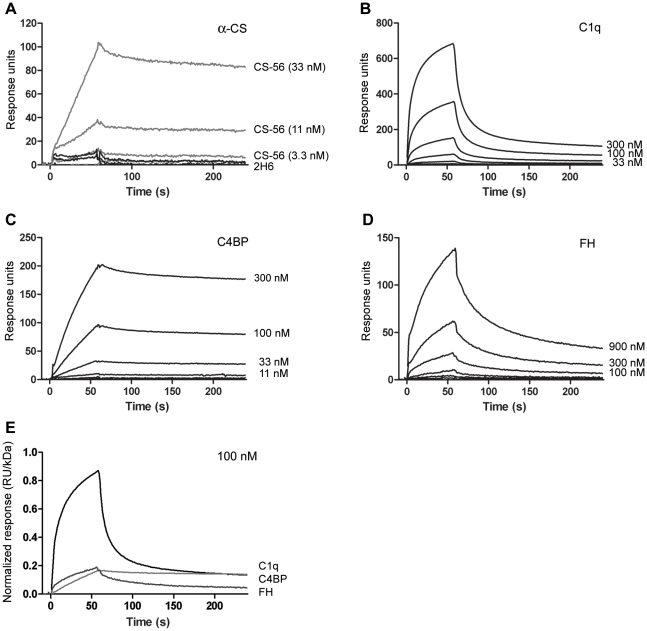
Surface plasmon resonance (SPR) analysis of complement proteins binding to immobilized CS-A. CS-A was immobilized to a biosensor chip and analyzed for binding of monoclonal antibodies (CS-56 and 2H6) (A), purified C1q (B), C4BP (C), and factor H (D) using SPR. Antibodies were tested from 33 nM; C1q and C4BP from 300 nM and factor H and C1INH from 900 nM, all in threefold dilutions. Binding response of C1q, C4BP and factor H at a constant concentration (100 nM), were normalized by dividing the response by the molecular mass of the analyte (E). Data are shown as mean, n = 3.

The interaction profiles of C1q, C1INH, C4BP and factor H were further studied by surface plasmon resonance analysis (Fig. 2). CS-A was immobilized to a high density on a carboxylated HC500 sensor chip, and purified proteins and monoclonal antibodies against CS-A were passed over the surface. Of the two analyzed antibodies, only CS-56 but not 2H6 showed specificity against immobilized CS-A. Whereas C1INH showed no significant binding even at the highest concentration (data not shown), specific protein interactions with CS-A were found for C1q, C4BP and factor H, thereby confirming the affinity chromatography results. Normalization of the binding responses by molecular weight revealed C1q as the strongest binder, while C4BP and factor H showed similar yet lower relative intensities at clearly distinct kinetics. Although kinetic rate constants could be extracted for all three complement proteins (Supplementary Figure S1 and Table S1), the absolute values have to be regarded with care due to the rather high surface density of CS-A, its potential structural heterogeneity, and the multivalency of its interaction with C1q and C4BP. C1q showed a very fast association phase but also a rather rapid dissociation phase. In contrast, complex formation with C4BP appeared to be much slower. However, once formed, the C4BP/CS-A complex was found to be highly stable. Finally, factor H showed intermediate to rapid rates for both the association and dissociation phase.

### Binding of complement components to activated platelets and blocking of the binding to CS-A with anti CS-A mAbs

**Figure 3:**
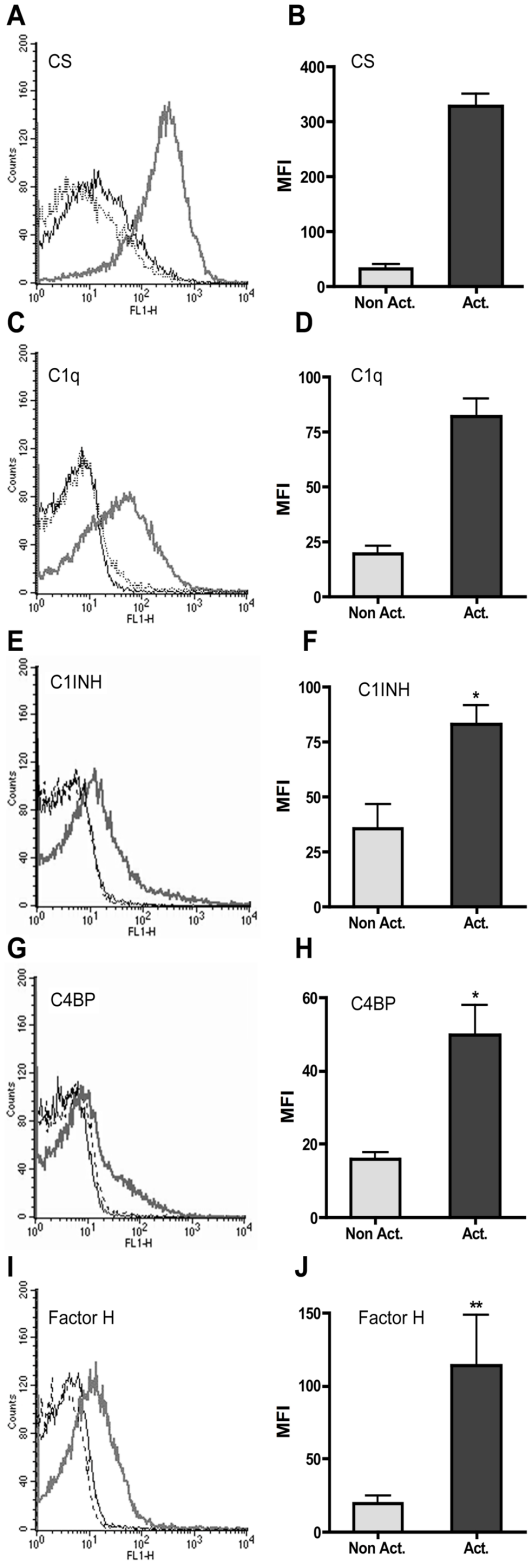
Exposure of CS-A and binding of complement components on TRAP-activated platelets. Exposure of CS-A (n = 3)(A, B) and binding of complement components C1q (n = 3)(C, D), C1INH (n = 5)(E, F), C4BP (n = 5)(G, H), and factor H (n = 8)(I, J) to non-activated (black line) and TRAP-activated platelets (gray line) in lepirudin-PRP, as detected by flow cytometry. Dotted lines indicate the binding of species-matched negative control Abs to activated platelets: sheep anti-mouse Ig (A, E, G, I) and rabbit anti-mouse Ig (C). The difference in binding between the nonactivated and activated samples was statistically significant (p<0.05). The flow cytometry figures shown are representative of those obtained for each of the bound components. * = p<0.05; ** = p<0.01.

Platelets expose CS-A on their surfaces after activation by thrombin, ADP, or other platelet activators. Exposure of CS-A was monitored using two anti-CS-A mAbs recognizing different epitopes of CS-A; mAbs CS-56 (Fig. 3A, B), and 2H6 (data not shown). Binding of 2H6 to TRAP-activated platelets gave a mean value of ∼60 mean fluorescence intensity (MFI) units; the binding of CS-56 was about five times higher. Binding of C1q (Fig. 3C, D) to TRAP-activated platelets in platelet rich-plasma (PRP) was also monitored in parallel to CS-A exposure on the platelets.

In order to investigate whether complement inhibitors bound to the surface of activated platelets, non-activated, and TRAP-activated platelets were subject to flow cytometry analyses. Platelets in lepirudin anti-coagulated PRP were activated with 33.5 µM TRAP. In non-activated PRP, no binding of complement inhibitors was observed when compared to the control using non-specific antibody. In contrast, C1INH (p<0.05), C4BP (p<0.05), and factor H (p<0.01)(Fig. 3E–J) bound to TRAP-activated platelets. The binding of the complement inhibitors was also confirmed using purified proteins and washed activated platelets (data not shown); however, no binding of C1INH was detected when washed, TRAP-activated platelets and purified protein were used.

**Figure 4:**
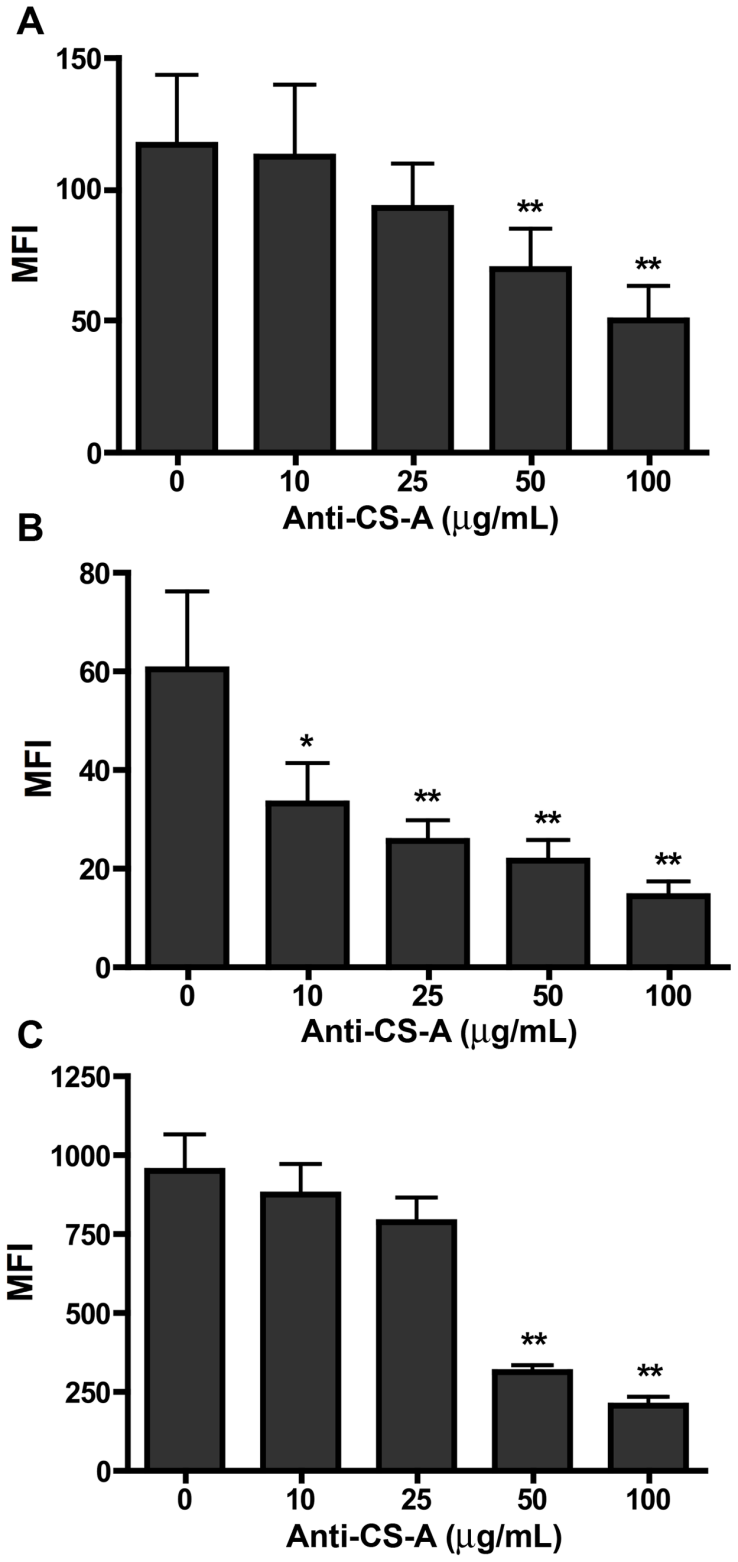
Inhibition of the binding of complement components to platelets by an anti-CS-A mAb. Platelets were preincubated with anti-CS-A mAb CS-56 in the indicated concentrations. After washing, C1q (n = 5)(A), C4BP (n = 6)(B), or factor H (n = 4)(C) was added, and the bound protein was detected using the appropriate antibody. Data shown as means ± SEM, * = p<0.05; ** = p<0.01.

The binding of C1q, C4BP, and factor H was inhibited by increasing concentrations of anti-CS-A mAb CS-56 (Fig. 4) but not by mAb 2H6 (data not shown). The C4BP binding to the platelet surface was significantly inhibited already with the lowest tested antibody concentration (10 µg/mL; approximately 50% inhibition). To significantly inhibit also binding of C1q and factor H, 50 µg/mL was needed (50% and 70% inhibition, respectively).

### Binding of HAGG to CS-A via C1q

**Figure 5:**
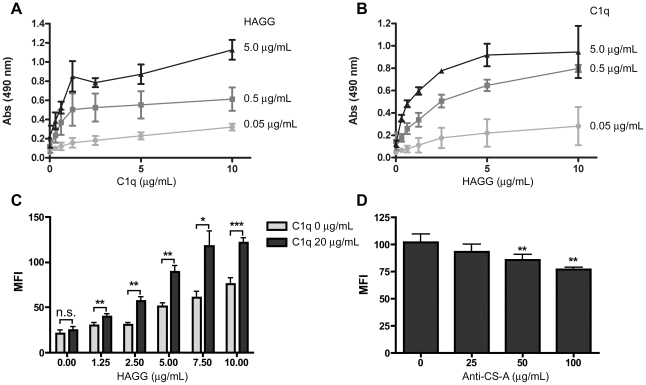
HAGG binding to immobilized CS-A and to the surface of activated platelets. C1q-dependent binding of HAGGs to CS-A. HAGGs were incubated with varying concentrations of C1q in microtiter plates bearing immobilized CS-A (A–B). HAGG in constant concentration, with C1q in dilution series (A) and C1q in constant concentration, with that of HAGG in dilution series (B). Binding of HAGGs to CS-A increased with increasing concentration of C1q. Binding of HAGGs at the indicated concentrations to washed activated platelets with and without the addition of C1q (C). The binding of HAGGs was inhibited by incubating washed TRAP-activated platelets with increasing concentrations of anti-CS-A mAb CS-56 prior to the addition of C1q and HAGG (D). Data shown are means ± SEM, (n = 3 (A–B), n = 5 (C–D); * = p<0.05; ** = p<0.01; *** = p<0.001).

In previous studies, it has been demonstrated that C1q can act as an Fc receptor for IgG [18], [19]. It is well established that upon platelet activation, CS-A is rapidly exposed on the surface of the cells. In order to test whether CS-A-bound C1q can act as an immunoglobulin binding protein, CS-A immobilized in microtiter plates was allowed to interact with heat-aggregated gamma globulin (HAGG) in the presence and absence of C1q. We found that the binding of HAGG was substantially amplified in the presence of C1q (Fig. 5A, B). In similar experiments using platelets, it was confirmed that C1q significantly increased the binding of HAGG to the platelet surface, indicating that C1q can act as a receptor for IgG (Fig. 5C). This binding was inhibited by the anti-CS-A mAb CS-56 (Fig. 5D).

## Discussion

CS-A is the major GAG exposed on activated platelets. The present study was undertaken to determine whether CS-A contributes to the binding of plasma proteins to activated platelets. We have recently shown that C1q can be prevented from binding to polystyrene surfaces conjugated with CS-A and to the surface of activated platelets by soluble CS-A at very high concentrations (50 mg/mL) [15]. In order to identify which plasma proteins are potential candidates for binding to CS-A, we employed immobilized CS-A to pull out proteins with an affinity/avidity for CS-A from human serum.

CS-A conjugated to Sepharose was shown to bind a restricted number of plasma proteins. When serum was passed over the CS-A-conjugated and control Sepharose columns, immunoglobulin-derived chains (μ, α, γ, and κ) were found to bind to both columns. In contrast, C1q was identified as the major specific CS-A-binding protein by SDS-PAGE, followed by mass spectrometry analysis and/or Western blotting. The affinity/avidity of C1q was superior to those of the other proteins. The binding of C1q to CS-A was independent of the presence of bound IgG, since depletion of IgG from serum did not change the amounts of C1q that bound to CS-Sepharose. This rules out that the immunoglobulins are natural, polyreactive, antibodies against CS-A or Sepharose, which potentially could bind C1q. Instead, it is likely that the bound immunoglobulins are bound due to charge interactions which is supported by the finding that there were more IgG than IgM, resembling the normal proportions of the IgG and IgM concentrations in serum/plasma, while most natural antibodies belong to the IgM class [20].

SPR analyses further confirmed these findings, demonstrating a high avidity for C1q binding to CS-A conjugated to the sensor chip. Also, C4BP and factor H bound specifically to CS-A on the sensor chip but at a lower response/molecular weight ratio than that of C1q, further supporting C1q's higher affinity for CS-A than that of C4BP or factor H. In contrast to C1q and factor H, C4BP demonstrated a slow dissociation from CS-A, revealing a rather high stability of the complexes once they had formed.

The strong affinity of C1q for CS-A indicates a multivalent binding to CS-A. C1q contains six collagenous stalks with globular heads [21]. In a previous study we have demonstrated that C1q binds to CS-A via the globular heads since we were able to block this interaction by the monoclonal antibody Anti-C1q-85 which is specific for the globular heads [15], [22]. The affinity of a single globular head for CS-A is not known, but that for IgG is in the range of 10^−3^ M [23]. Assuming that the affinity for CS-A is similar, the avidity (i.e., the combined affinities of the each of the engaged globular heads in binding to CS-A) is very strong, explaining the extremely high binding capacity of C1q for CS-A.

We also asked whether three complement proteins, all of which are known to interact with different GAGs on activated platelets, are exposed on the surface of TRAP-activated platelets in our system. Exposure of C1q and factor H on activated platelets has previously been documented [24], [25], [26], and factor H has also been demonstrated on non-activated platelets. We have also shown that C4BP is present on the activated platelet surface. In order to link the binding of these proteins to exposure of CS-A, we treated platelets with chondroitinase. This treatment led to loss of CS-A, but the proteins and mAbs still bound to the surface of activated platelets (data not shown). Obviously, there were still CS-A remnants left, or else there is continuous recruitment of CS-A to the surface of activated platelets. Instead, we used the CS-A-specific mAbs CS-56 and 2H6 to block the binding of the three candidate proteins. CS-56 and 2H6 bind to different distinct epitopes of CS-A, which are present to various degrees depending on the origin of CS-A [27]. CS-56, but not 2H6, showed specific binding to immobilized CS-A as well as CS-A present on the platelet surface. Preincubating CS-56 with platelets led to a significant inhibition of the binding of C1q, C4BP, and factor H to activated platelets.

Receptors and binding proteins for C1q, C4BP, and factor H on platelets have previously been described. In the case of C1q, several receptors have been identified that either recognize the globular heads (e.g. gC1qR) or the collagenous portion (e.g. cC1qR) [25], [28], [29]. C4BP has been shown to bind to membrane-bound protein S [30], and factor H has been shown to interact with GPIIb/IIIa [31]. The fact that mAb CS-56 was able to block the binding of all three proteins to platelets indicates that CS-A indeed represents a binding molecule on the surface of activated platelets. Whereas C1q and factor H were only partially blocked by mAb CS-56 and may likely have additional sites on platelets, the observation that C4BP binding was almost completely inhibited by this antibody suggests that CS-A is the primary interaction site for C4BP on activated platelets.

C1q has previously been shown to be involved in the binding of immune complexes to activated platelets [19], [32]. In order to confirm these reports, we allowed C1q to bind to CS-A in microtiter plates, followed by exposure to HAGG. A pronounced amplification of the binding of HAGG to the surfaces was observed. This result clearly demonstrated the involvement of C1q in the binding of HAGG to immobilized CS-A, bridging the immune complex to the surface. Despite the obvious involvement of several of the globular heads in binding to CS-A, sufficient binding sites were present to bind HAGG in the microtiter plate assay. In similar experiments, C1q and HAGG were allowed to bind to activated platelets. A two-fold increase in the binding of HAGG to activated platelets was obtained, confirming that C1q acts as an immunoglobulin binding protein on platelets.

In order to connect the binding of HAGG to the CS-A-mediated binding of C1q, we attempted to block the binding by using a specific mAb against CS-A. A clear inhibition was obtained, further linking the binding of C1q to CS-A with the binding of HAGG. However, these experiments do not address any possible involvement in the collagenous section of C1q in the interaction with platelets.

Previous studies have demonstrated that the binding of immune complexes to platelets has the capacity to activate platelets. Thus, the binding of C1q to platelets is of potential pathological importance for immune complex diseases such as SLE and vasculitides. During exacerbations of these diseases, the levels of immune complexes rise, resulting in binding of immune complexes and activation of platelets [19], [32]. This is indeed the case in SLE, in which platelet activation is a common sign of disease exacerbation.

## Materials and Methods

### Ethics statement

This study was approved by the Medical Ethical Committees at the Universities of Linköping, and Uppsala, Sweden, and written informed consent was given by the blood donors.

### Blood samples

Blood was drawn from healthy volunteers who had received no medication for at least 10 days prior to donation. It was collected in 7-mL vacutainer tubes containing 100 µL of 5 mg/mL lepirudin (Shering AG, Saksa, Germany). To obtain PRP, blood was centrifuged at 150×g for 15 min at room temperature (RT) within 30 min of collection.

### Purified complement proteins

Human C1q was prepared from human serum as described by Tenner et al. [33], and factor H was prepared from human serum essentially according to Hammer et al. [34], except that the first step consisted of a euglobulin precipitation as described by Nilsson and Műller-Eberhard [35]. Purified C4BP and C1INH were purchased from Complement Technology, Inc. (Tyler, TX, USA).

### Preparation of a CS-A affinity column

A CS-A affinity column (15-mL bed volume) was generated by covalent coupling of CS-A via primary amines in the protein core to CNBr-activated Sepharose™ 4B (GE Healthcare Bio-Sciences AB, Uppsala, Sweden). The Sepharose was prepared according to the manufacturer's protocol. CS-A (450 mg), dissolved in 45 mL coupling buffer (0.1 M sodium bicarbonate buffer, pH 8.3, containing 0.5 M NaCl), was added to the prepared Sepharose (4500 mg) and allowed to react with agitation for 2.5 hours. Any remaining reactive groups on the Sepharose were blocked by overnight incubation at 4°C with 0.1 M Tris-HCl, pH 8.0. Ionically bound CS-A was removed from the Sepharose by repeated washing steps of alternating 0.1 M acetate buffer, pH 4.5, containing 0.5 M NaCl and coupling buffer. A column prepared using coupling buffer that was lacking CS-A was used as a control. CS-A-affinity and control Sepharose preparations were packed in glass econo columns (Bio-Rad, Hercules, CA, USA) using gravity flow and equilibrated in 20 mM phosphate buffer pH 7.4 containing 0.075 M NaCl and 10 mM EDTA.

### Application of human serum or C1q-depleted serum to the affinity column

Serum obtained from the Division of Clinical Immunology and Transfusion Medicine, Uppsala University Hospital, was depleted of C1q (99% depletion) as previously described [36]. Serum or C1q-depleted serum was diluted 1∶3 in 20 mM phosphate buffer pH 7.4 containing 0.075 M NaCl with 10 mM EDTA, and loaded in equal amounts on CS-A and control column (45 mL of diluted serum per column). Columns were washed with 75 mL of 20 mM phosphate buffer pH 7.4 containing 0.075 M NaCl and 10 mM EDTA. The bound proteins were eluted in 1-mL fractions with 20 mM phosphate buffer pH 7.4 containing 1.0 M NaCl and 10 mM EDTA. The amount of eluted protein in each fraction was estimated by absorbance measurements at 280 nm. In both cases, eight fractions with the highest protein content were pooled and concentrated to 500 µL using Amicon Ultra Centrifugal Filters (10-kDa cut-off) (Millipore AB, Sundbyberg, Sweden). IgG dependent binding of C1q to CS-A was investigated by loading intact serum and serum which had been depleted of IgG using a HiTrap protein G sepharose column (GE Healthcare) on CS-A Sepharose. The amount of C1q in the eluate was measured by ELISA as previously described [37].

### SDS-PAGE and Western blotting

Concentrated proteins from the CS-A and control column were separated by SDS-PAGE under reducing conditions. Proteins were either detected with Coomassie brilliant blue or transferred to PVDF membranes (Bio-Rad), then blocked with 1% bovine serum albumin (BSA). The membranes were incubated with rabbit anti-C1q (Dako, A/S Glostrup, Denmark), goat anti-C1INH (Enzyme Research Laboratories, South Bend, IL, USA), rabbit anti-C4BP (The Binding Site, Birmingham, UK), or sheep anti- factor H (The Binding Site), all of which were biotinylated. Membranes were thereafter incubated with HRP-conjugated streptavidin (GE Healthcare) and developed with diaminobenzidine (Sigma-Aldrich Inc, St. Louis, MO, USA).

### In-gel digestions

Proteins that had been separated by SDS-PAGE and visualized with Commassie brilliant blue staining were subjected to in-gel digestion and identified by either MALDI-MS/MS or LC-ESI-MS/MS. In-gel digestion was performed according to UCSF in-gel digestion protocol (http://donatello.ucsf.edu/ingel.html; accessed April 27, 2010). In brief, excised bands were dehydrated with 25 mM NH_4_HCO_3_ in 50% acetonitrile, reduced with 10 mM DTT at 56°C for 60 minutes and alkylated with 55 mM iodoacetamide at RT for 45 minutes in the dark. Proteolytic cleavage was carried out by over night incubation with trypsin (sequencing grade modified trypsin (16 ng/µL), Promega, Madison, WI, USA) at 37°C. Peptides were then extracted by the addition of 50% acetonitrile containing 5% formic acid. The solvent was evaporated to dryness in a vacuum concentrator and the extracted peptides were redissolved in 0.1% trifluoroacetic acid (TFA). When necessary, the samples were desalted using ZipTip_C18_ (Millipore, Bedford, MA, USA).

### Mass spectrometry and protein identification

Mass spectrometric analysis of in-gel digests was performed using a SYNAPT HDMS (Waters Corp., Milford, MA, USA) controlled by MassLynx software v 4.1 (Waters) and equipped either with a MALDI or nanoESI source. A nanoACQUITY UPLC (Waters) system was used for peptide separation by reversed-phase liquid chromatography. After injection, peptides were trapped for 3 min with 3% mobile phase A at 5 µL/min on a 5 µm Symmetry C18 column (180 µm×20 mm, Waters) and further separated on a 1.7 µm BEH130 C18 column (75 µm×150 mm, Waters). The analytical column temperature was held at 35°C. Mobile phase A was 0.1% formic acid in water and B was 0.1% formic acid in acetonitrile. Peptides were separated with a 40 min gradient (3–40%) at flow rate 0.3 µL/min and eluted into the nanoESI source. The capillary voltage was 3.2 kV, the cone voltage was 37 V and the source temperature was set to 100°C. [Glu^1^]-fibrinogen peptide was used for lock-mass correction with a sampling rate of 30 s. Mass spectra were acquired in positive mode and data dependant analysis was used for selection of precursors. MS survey data was acquired over a m/z range 400–2000 at scan rate 0.6 s. MSMS of ions with charge +2, +3 or +4 was obtained by collision energy ramp from 15 to 40 V over a m/z range of 50–2000. The dynamic exclusion window was set to 60 s. For MALDI-MS/MS analysis 1 µL of sample was mixed with 1 µL of matrix solution (saturated CHCA in 50% ACN, 0.1% TFA) on the target plate. Mass spectra were acquired in positive mode over the mass range m/z 700–3500 at scan rate 2 s. Three precursors showing peak intensity threshold above 15 counts were selected for fragmentation and the MS/MS spectrum was acquired between m/z 50 and the precursor mass. Data processing and analysis was carried out by PLGS (Waters) software version 2.3. Processed data was searched with Mascot (matrix science) search engine against Swiss-Prot database with the following parameters: human taxonomy; 20 ppm precursor mass tolerance; 0.25 Da fragment ion tolerance; trypsin enzyme; one missed cleavage allowed; carbamidomethylation of cysteine and oxidation of methionine as variable modifications. Protein identification criteria were based on Mascot score above the threshold (p<0.05) and a minimum of one peptide fragmentation spectrum.

### Interaction analysis of CS-A with complement proteins using surface plasmon resonance

Surface plasmon resonance (SPR) analysis was performed on a Biacore X biosensor (Biacore AB, Uppsala, Sweden) with CS-A immobilized onto a carboxylated hydrogel sensorchip (HC500; XanTec bioanalytics GmbH, Munster, Germany). The sensor chip was conditioned and activated for 10 minutes using sulfo-NHS amine coupling kit (XanTec) according to the manufacturers' protocol. A 50 µL injection of CS-A (100 µg/mL) dissolved in 20 mM phosphate buffer pH 7.0 was performed in one flow cell followed by quenching with 1 M ethanolamine hydrochloride pH 8.5, whereas the other flow cell served as control and was quenched by ethanolamine after activation. Approximately 600 RU of CS-A was immobilized, and the successful immobilization was verified by an anti-CS-A mAb (CS-56; Sigma-Aldrich). All interaction studies were performed in HBS buffer (10 mM HEPES pH 7.4, 150 mM NaCl, 0.005% Tween-20) at 20°C and a flow rate of 20 µL/min. Purified proteins C1q (1.2–300 nM), C4BP (1.2–300 nM), C1INH (3.7–900 nM), factor H (3.7–900 nM) as well as antibodies CS-56 (1.2–33 nM) and 2H6 (Seikagaku Corp. Tokyo, Japan; 1.2–33 nM), which recognize different epitopes of CS-A [27], were analyzed as threefold dilution series. Each analyte concentration was injected in triplicates for 60 s with a dissociation time of 180 s. The sensor surface was regenerated using repetitive injections of 1 M NaCl in 50 mM sodium acetate buffer pH 4.5 and/or 1 M NaCl in 0.1 M sodium borate pH 9.0. All sensorgrams were processed using the double referencing method [38] where background contribution and buffer artifacts are eliminated by subtracting signals from the reference flow cell and from buffer blank injections. Kinetic parameters were analyzed using ClampXP (version 3.5) [39]. The data for all binding proteins were fitted with the surface heterogeneity model, describing immobilized ligand (B) existing in different forms with different rate constants for interacting with the analyte, represented as equation 1 [40]. 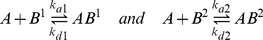
(1)


### Platelet activation and preparation

Platelets were activated in whole blood or PRP anticoagulated with lepirudin at 37°C by adding thrombin receptor activating peptide-6 (TRAP; (SFLLRN), Invitrogen, Molecular Probes, Carlsbad, CA, USA) to a final concentration of 25 µg/mL (33.5 µM). Activation was stopped by the addition of EDTA (10 mM). The PRP was centrifuged at 1100×g for 10 min at RT, and the plasma was removed.

After activation and centrifugation, the platelet-containing pellets were washed three times with Tyrode's medium I (pH 6.5), containing 137 mM NaCl, 2.7 mM KCl, 1 mM MgCl_2_, 0.36 mM NaH_2_PO_4_, 12 mM NaHCO_3_, 2 mM CaCl_2_, 5.5 mM glucose, 3.5 mg/ml BSA (Sigma-Aldrich), 1 µM PGE_1_ (Sigma-Aldrich), and 2 IU/mL heparin (Bioiberica, Barcelona, Spain). Each wash cycle consisted of suspension of the pellet in the Tyrode's medium and incubation at 37°C for 10 min, followed by centrifugation at 1100×g at RT for 10 min. In some experiments, the platelets were washed prior to activation. Platelets were pelleted from PRP by centrifugation at 1100×g for 10 min and washed three times as described above to remove plasma proteins. After washing, the platelets were pelleted and resuspended in Tyrode's medium I without the addition of BSA, heparin, or PGE_1_. The platelets were then activated by the addition of TRAP and incubated as described above. After activation, they were washed once and resuspended in Tyrode's medium I with BSA, heparin, and PGE_1_. Activated and non-activated platelets were diluted to 200×10^9^/L, and purified C1q (10 µg/mL), C1INH (20 µg/mL), C4BP (20 µg/mL), or factor H (20 µg/mL) was added to the platelets and incubated for 30 min at RT. In a few experiments, platelets were pre-incubated with anti-CS-A mAb CS-56 (Sigma-Aldrich), (10, 25, 50 or 100 µg/mL) on a shaker for 30 min at RT prior to the addition of C1q, C4BP, or factor H to the platelets. Platelets were then washed and labeled with antibodies as described below. In order to determine whether C1q bound to activated platelets could act as an Fc receptor for IgG, we added model complexes in the form of heat-aggregated gamma globulin (HAGGs; 0–10 µg/mL) to activated platelets that been incubated with or without C1q (10 µg/mL) followed by washing, then incubated the platelets and complexes for 30 min at RT. After washing, the binding of HAGG was detected with rabbit anti-human IgG (Fab_2_)-FITC (Dako). In a few experiments platelets were incubated with anti-CS-A mAb CS-56 (25, 50 and 100 µg/mL) prior to the addition of C1q (10 µg/mL) and subsequently HAGG (10 µg/mL).

### FACS analysis of platelet-bound complement proteins

Flow cytometry was used to monitor the activation of platelets and detect the binding of complement proteins to non-activated platelets or platelets activated with 33.5 µM TRAP. Platelets (100 µL, 50×10^9^ platelets/L) were incubated for 60 min at RT with the following antibodies: mouse anti-human P-selectin-RPE (Dako) or AlexaFluor 488-labeled rabbit anti-human C1q (Dako). Binding of the complement regulators was detected with biotinylated sheep polyclonal antibodies against human factor H, human C1INH, and human C4BP (The Binding Site). Exposure of chondroitin sulfate on TRAP-activated platelets was monitored with biotinylated IgM monoclonal anti-CS-A antibody 2H6 (Seikagaku Corp) or with IgM monoclonal anti-CS-A antibody CS-56 followed by sheep anti-mouse Ig-FITC. Streptavidin-FITC (GE Healthcare) was used to detect the bound biotinylated antibodies. FITC-labeled sheep anti-mouse Ig (The Binding Site), rabbit anti-mouse Ig (Dako), and mouse IgG1 (Dako), and biotinylated mouse IgG1 and goat anti-rabbit Ig were used as negative controls, each at a final concentration of 10 µg/mL. In each experiment, the activation status of the platelets (control and experimental) was ascertained by monitoring the expression of P-selectin. After staining, the platelets were washed and fixed with 0.2% paraformaldehyde (Apoteket, Gothenburg, Sweden). The bound fluorochrome-labeled antibodies were monitored with a FACSCalibur (Becton Dickinson, San Jose, CA, USA) and BD CellQuest Pro software. In each sample, ∼30,000 cells were analyzed.

### Biotinylation of CS-A and preparation of CS-A-coated microtiter plates

CS-A sodium salt (Sigma-Aldrich) was biotinylated via the primary amines in the protein core as follows: CS-A (20 mg) was dissolved in 0.1 M phosphate buffer pH 7.6 (2 mL), and 1.7 mg biotinamidohexanoic acid N-hydroxysuccinimide ester (Sigma-Aldrich) was added to the solution. The reaction was allowed to take place over 10 hr at 4°C. Unconjugated biotin and reaction byproducts were removed with a PD-10 desalting column (GE Healthcare).

Microtiter plates (Nunc Maxisorb Immunoplate, Nunc, Copenhagen, Denmark) were coated with 100 µL streptavidin (Sigma-Aldrich; 5 µg/mL). Wells were then blocked with 10 mM phosphate buffer containing 1% BSA, 0.05% Tween 20 (Sigma-Aldrich), and 0.145 M NaCl, for 30 min at RT. Biotinylated CS-A (100 µL, 5 µg/mL) was subsequently bound to the streptavidin layer by incubation for 60 min at RT.

### C1q-dependent binding of HAGG to immobilized CS-A

Purified C1q and HAGG were diluted in PBS. C1q was incubated in CS-A-coated wells at a constant concentration (0.05, 0.5, or 5 µg/mL) or in a two-fold dilution series (0.31–20 µg/mL) for 15 min at RT. HAGG at a constant concentration (0.05, 0.5, or 5 µg/mL) or in a two-fold dilution series (0.31–20 µg/mL) was added to the wells with the C1q in dilution series or a constant concentration, respectively, and incubated for 60 min at RT. After washing, bound HAGG was detected using rabbit anti-human IgG (Dako) followed by HRP-conjugated swine anti-rabbit antibody (Dako). Washing and dilution of the antibodies were done using PBS with 0.05% Tween-20. Phenylenediamine dihydrochloride in 0.1 M citrate, pH 5, was used as the color substrate.

### Statistical analysis

Results are presented as means ± SEM. Differences between means were statistically evaluated using the paired Student's t-test or repeated measures ANOVA, followed by Bonnferroni's multiple comparison test for calculating differences in data containing more than two variables. For statistical analysis, Prism 4 for Macintosh software (Graphpad, San Diego, CA, USA) was used. In the experiments in which platelets were used, the n-values refer to independent experiments using platelets from different blood donors.

## Supporting Information

Table S1 Binding parameters of C1q, C4BP and factor H to immobilized CS-A.
(0.03 MB DOC)

Figure S1 Sensorgrams fitted to the surface heterogeneity model using ClampXP software. CS-A immobilized to a biosensor chip and analysed for binding of purified C1q (1.2–300 nM; A), C4BP (1.2–300 nM; B), and factor H (3.7–900 nM; C) using SPR. Data are shown as mean, n = 3. C1q sensorgrams representing 100 and 300 nM were, based on the high response, excluded in the final fitting. Experimental data are shown as dashed black lines and fit as solid grey lines. Sensorgrams were fitted using heterogeneity model, describing different form of immobilized ligand able to interact with the analyte with separate rate constants. This assumption is due to the structural diversity that exists among CS-A.
(0.24 MB TIF)
