# Supplementary material for: Contribution of Chondroitin Sulfate A to the Binding of Complement Proteins to Activated Platelets
Source: PLoS One. 2010 Sep 23;5(9):e12889. doi: 10.1371/journal.pone.0012889 (PMC2944812; doi:10.1371/journal.pone.0012889)
Supplement: Table S1 — Binding parameters of C1q, C4BP and factor H to immobilized CS-A. (0.03 MB DOC) [file pone.0012889.s001.doc]

**Supplemental Table S1**. Binding parameters of C1q, C4BP and factor H to immobilized CS-A.

| Protein | *k_a1_* (M^-1^ s^-1^) | *k_d1_* (s^-1^) | *k_a2_* (M^-1^ s^-1^) | *k_d2_* (s^-1^) | K_D1_ (nM) | K_D2_ (nM) |
| --- | --- | --- | --- | --- | --- | --- |
| C1q | 9.02 x 10^5^ | 9.81 x 10^-2^ | 2.04 x 10^5^ | 4.02 x 10^-3^ | 109 | 19.8 |
| C4BP | 5.82 x 10^4^ | 2.26 x 10^-4^ | 2.04 x 10^5^ | 1.75 x 10^-2^ | 3.97 | 86.1 |
| Factor H | 1.06 x 10^5^ | 8.81 x 10^-2^ | 2.43 x 10^4^ | 4.97 x 10^-3^ | 834 | 204 |

Association (*k_a1_*, *k_a2_*) and dissociation (*k_d1_*, *k_d2_*) rate constants derived from sensorgrams fitted to the surface heterogeneity model using ClampXP software. The dissociation constants (K_D1_, K_D2_) are determined as *k_d1_*/*k_a1_* and *k_d2_*/*k_a2_* respectively. The absolute values have to be regarded with care due to the rather high surface density of CS-A, its potential structural heterogeneity, and the multivalency of its interaction with C1q and C4BP.
