# Supplementary figures and images for: Contribution of Chondroitin Sulfate A to the Binding of Complement Proteins to Activated Platelets
Source: PLoS One. 2010 Sep 23;5(9):e12889. doi: 10.1371/journal.pone.0012889 (PMC2944812; doi:10.1371/journal.pone.0012889)

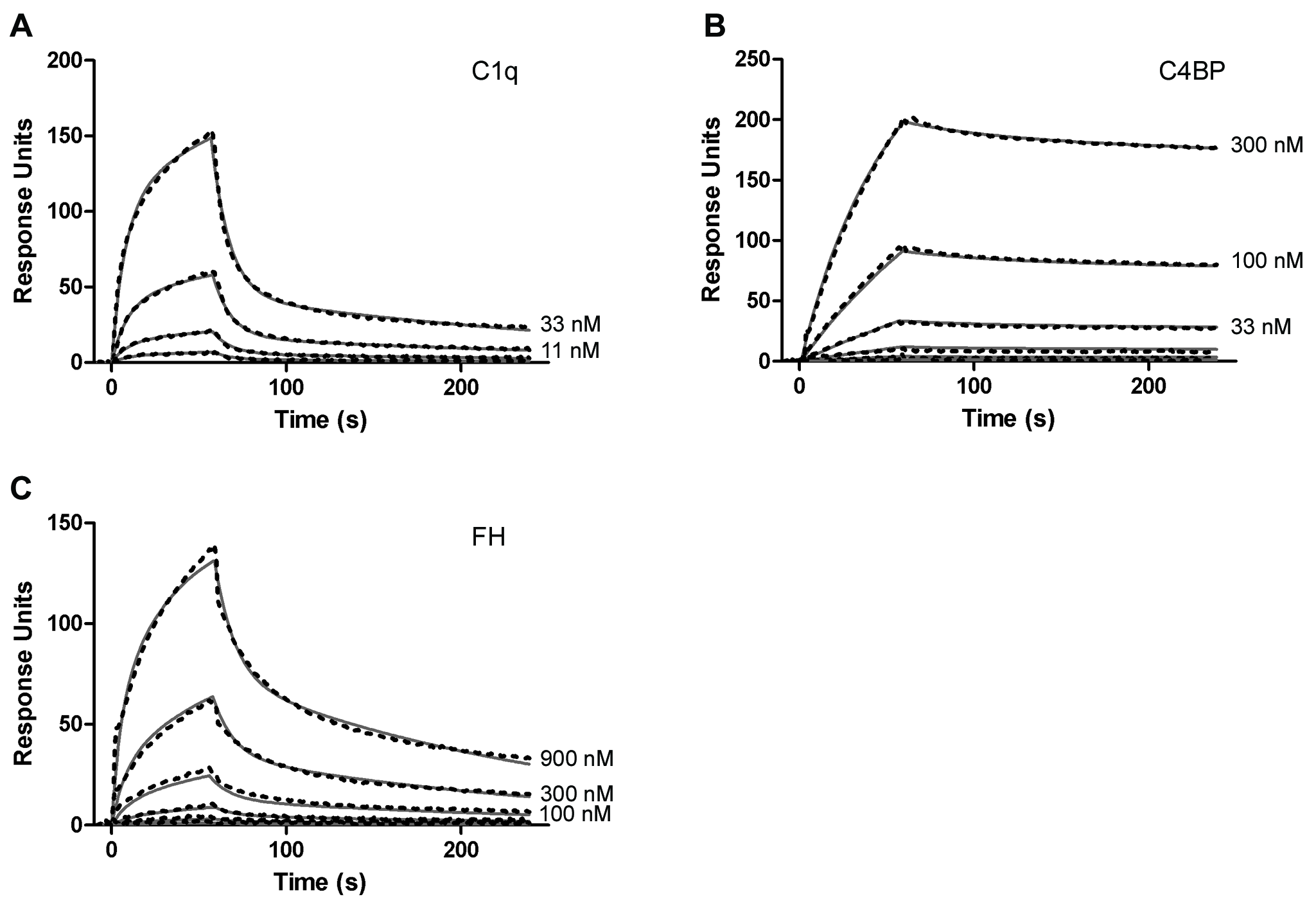

Supplement: Figure S1 — Sensorgrams fitted to the surface heterogeneity model using ClampXP software. CS-A immobilized to a biosensor chip and analysed for binding of purified C1q (1.2–300 nM; A), C4BP (1.2–300 nM; B), and factor H (3.7–900 nM; C) using SPR. Data are shown as mean, n = 3. C1q sensorgrams representing 100 and 300 nM were, based on the high response, excluded in the final fitting. Experimental data are shown as dashed black lines and fit as solid grey lines. Sensorgrams were fitted using heterogeneity model, describing different form of immobilized ligand able to interact with the analyte with separate rate constants. This assumption is due to the structural diversity that exists among CS-A. (0.24 MB TIF) [file pone.0012889.s002.tif]
